# Are Rotations and Translations of Head Posture Related to Gait and Jump Parameters?

**DOI:** 10.3390/jcm12196211

**Published:** 2023-09-26

**Authors:** Nabil Saad, Ibrahim M. Moustafa, Amal Ahbouch, Nour Mustafa Alsaafin, Paul A. Oakley, Deed E. Harrison

**Affiliations:** 1 Department of Physiotherapy, College of Health Sciences, University of Sharjah, Sharjah 27272, United Arab Emiratesiabuamr@sharjah.ac.ae (I.M.M.);; 2 Neuromusculoskeletal Rehabilitation Research Group, RIMHS–Research Institute of Medical and Health Sciences, University of Sharjah, Sharjah 27272, United Arab Emirates; 3 Kinesiology and Health Sciences, York University, Toronto, ON M3J 1P3, Canada; 4 Independent Researcher, Newmarket, ON L3Y 8Y8, Canada; 5 CBP Nonprofit (a Spine Research Foundation), Eagle, ID 83616, USA

**Keywords:** head posture, biomechanical parameters, sports performance, posture, gait, jump

## Abstract

This study assessed the relationship between head posture displacements and biomechanical parameters during gait and jumping. One hundred male and female students (20 ± 3 yrs) were assessed via the PostureScreen Mobile® app to quantify postural displacements of head rotations and translations including: (1) the cranio-vertebral angle (CVA) (°), (2) anterior head translation (AHT) (cm), (3) lateral head translation in the coronal plane (cm), and (4) lateral head side bending (°). Biomechanical parameters during gait and jumping were measured using the G-Walk sensor. The assessed gait spatiotemporal parameters were cadence (steps/min), speed (m/s), symmetry index, % left and right stride length (% height), and right and left propulsion index. The pelvic movement parameters were (1) tilt symmetry index, (2) tilt left and right range, (3) obliquity symmetry index, (4) obliquity left and right range, (5) rotation symmetry index, and (6) rotation left and right range. The jump parameters measured were (1) flight height (cm), (2) take off force (kN), (3) impact Force (kN), (4) take off speed (m/s), (5) peak speed (m/s), (6) average speed concentric phase (m/s), (7) maximum concentric power (kW), (8) average concentric power (kW) during the counter movement jump (CMJ), and (9) CMJ with arms thrust (CMJAT). At a significance level of *p ≤* 0.001, moderate-to-high correlations (0.4 < *r* < 0.8) were found between CVA, AHT, lateral translation head, and all the gait and jump parameters. Weak correlations (0.2 < *r* < 0.4) were ascertained for lateral head bending and all the gait and jump parameters except for gait symmetry index and pelvic symmetry index, where moderate correlations were identified (0.4 < *r* < 0.6). The findings indicate moderate-to-high correlations between specific head posture displacements, such as CVA, lateral head translation and AHT with the various gait and jump parameters. These findings highlight the importance of considering head posture in the assessment and optimization of movement patterns during gait and jumping. Our findings contribute to the existing body of knowledge and may have implications for clinical practice and sports performance training. Further research is warranted to elucidate the underlying mechanisms and establish causality in these relationships, which could potentially lead to the development of targeted interventions for improving movement patterns and preventing injuries.

## 1. Introduction

Gait and jump analysis hold immense importance for understanding the biomechanics and functional abilities of athletes across all sports. These analyses provide valuable information about the quality and efficiency of movement patterns, offering insights into musculoskeletal health, performance, and injury prevention [1,2,3,4,5,6,7]. Understanding an individual’s jumping and gait mechanics is crucial, particularly when considering the spinal factors that may affect these parameters. One such factor is head posture, which is proposed to play a significant role in the overall relationship between movement patterns and spinal alignment [8,9,10,11,12,13]. It is plausible that alterations in gait and jump performance are associated with abnormal head posture; this hypothesis stems from the understanding that deviations in head posture can detrimentally affect both postural control and proprioception [9,14,15,16]. Consequently, the effects of abnormal head/neck postures can have a cascading impact on the entire kinetic chain, resulting in modifications in gait and jump parameters, as well as an elevated susceptibility to injuries through alterations of balance and proprioception [17,18,19,20]. Despite the significance of this potential relationship, limited research has been conducted to investigate the connection between head posture and the mechanics of gait and jump movements, and research has been limited primarily to the forward head posture (FHP) [12]. For example, in a recent systematic review, it was identified that “current evidence supports an association between FHP and a detrimental alteration in limits of stability, performance-based balance, and cervical proprioception”.

Importantly, since posture is three-dimensional (3D), it should be assessed in three-dimensions, however, most previous studies have only focused on sagittal plane alignment and have not considered coronal plane (lateral) translational and rotational postural differences [12,17,18,21,22]. Further, to the best of our knowledge, there are currently no studies that have investigated this relationship within the context of translational and rotational head posture measurement differences specifically among university students. Biomechanically, the movements of the spine are intricate and involve complex coupling patterns that are influenced by the anatomical and mechanical characteristics between two or more motion segments [23,24,25,26]. A primary main motion as a translation or rotation of the head/cervical spine in one geometric plane can lead to “coupled” movements in other planes [27,28]. This emphasizes the importance of conducting a global posture assessment that considers translational and rotational displacements, as suggested by Harrison and Oakley [29,30]. As postural displacements can exist in multiple planes, there is a need for a more comprehensive understanding of the relationship between 3D posture parameters and gait parameters. Upon reviewing evidence, however, the relationship between posture and gait/jump biomechanics has not been sufficiently covered, as most measures of posture that are correlated with gait/jump analysis have not been assessed in 3D [31,32,33,34], and only limited research has been performed on the sagittal cervical spine [12].

Recent technological advancements have made it possible to accurately measure posture parameters, including translational and rotational displacements in multiple planes [35,36]. These advancements present a unique opportunity to identify potential areas of postural abnormalities for interventions that can improve gait and jump parameters across various population groups due to their accessibility, accuracy, and ease of use. In the context of analyzing gait and jump performance, the current gold-standard laboratory-based assessment methods, such as motion capture systems, optical encoders, position transducers, and force plates offer high accuracy but are limited in their clinical use due to setup and analysis time, technical expertise requirements, and high costs. To overcome these limitations, the valid and reliable wireless inertial BTS G-WALK sensor system (G-Walk) has been introduced as a more practical alternative for gait and jump performance assessment [37,38]. Thus, the aim of this study is to provide a more comprehensive understanding of the relationship between 3D head/neck posture parameters and gait and jump parameters that are essential to understanding the effects of 3D postural parameters on performance during walking and jumping across healthy collegiate students. The current study tests the main hypothesis that posture displacements as rotations and translations of the head will have negative impacts on gait and jumping in a young, healthy student population.

## 2. Materials and Methods

For this study, one hundred healthy male and female collegiate students were recruited for participation. The study followed a cross-sectional observational design where all measurements were taken in one sitting. The inclusion criteria included (i) ages between 17 and 26; (ii) a normal body mass index (BMI) of up to 24.9; and (iii) no previous history of musculoskeletal/movement disorders of any kind. Ethical approval was obtained from the Ethics Committee of the University of Sharjah; reference number: REC-21-10-25-S. Informed consent was also obtained from all the participants prior to data collection in accordance with relevant guidelines and regulations. All participants were screened for the following exclusion criteria: (i) inflammatory joint disease or other systemic pathologies; (ii) prior history of overt injury and surgery relating to the musculoskeletal system or disorder related to the spine and extremities; (iii) musculoskeletal pain in the previous three months; (iv) neurological disorders; and (v) vision and/or hearing related impairments.

### 2.1. Outcome Measures

#### 2.1.1. Posture Measurement 

The PostureScreen^®^ Mobile app (PSM) is a digital posturographic assessment tool that was used to perform a 3D postural examination. The PSM has been established as a reliable and valid method for evaluating static posture [35,36]. For example, investigations have identified that PSM has an intra-rater reliability that ranges from 0.71 to 0.99, and an inter-rater reliability which is good to excellent for all translations (ICC’s between 0.85 and 0.98) [35,36]. PSM captures images of the participant from four directions: anterior and posterior (coronal plane) and the left and right sides (sagittal plane). After the photographic capture, specific anatomical reference points are digitized by the user such as the pelvic iliac spines, the greater trochanter, the femoral condyle, and the tragus. To ensure maximum accuracy of the manual digitization of landmarks, participants were instructed to undress/wear clothing that exposes the landmarks required so that they could be identified and labelled prior to digitization. Moreover, the landmarks were digitized by the same research team member and then cross-checked by the same 2 members to ensure accuracy for all participants’ data. The PSM then calculates specific body angles and distances based on the anatomical digitization and creates an output file containing values of posture variables and images of the participant that can be used to compare and analyze the postural deviations from neutral among participants.

The following postural parameters were assessed:

- Cranio-vertebral angle (CVA), which is the acute angle that is formed between a straight line that connects the spinous process of C7 to the tragus of the ear, and the horizontal line that passes through the spinous process of C7.
- Anterior head translation, which is the movement of the head anteriorly.
- Lateral head translation in the coronal plane, which is movement of the entire head to either side.
- Lateral head side bending, which is bending the head towards either side.

#### 2.1.2. Gait and Jump Parameters 

The G-Walk is a portable gait lab system that functions using a special sensor which is placed on each participant. The sensor can provide objective data regarding kinetics, kinematics, and spatio-temporal parameters by accurately measuring components of movement in 6 integrated protocols, 2 of which were utilized in this study [39,40]. The G-walk system has been used in several studies for gait and jump analysis [41,42]. Further, the G-Walk system can differentiate between parameters for the left and right sides, in addition to comparing values obtained with established normal ranges based on each participant’s sex, age, height, and weight [41,42].

The G-Walk measurement includes several protocols. This study mainly explores the data related to two protocols, one of which is related to ambulation, called “walking”, and the other protocol is related to athletic performance, called “jumping”. Each protocol measures certain pre-set parameters related to kinetics, kinematics, spatio-temporal parameters, and specific general parameters associated with that activity. Moreover, the jumping protocol includes several types of jumps, of which two were selected for this study. Regarding the selection of the walking and jumping protocols, these two tasks were primarily chosen because they represent two important aspects of functional capability. Regarding the jumping protocol, the countermovement jump (CMJ) was selected because it is a simple, practical, valid, and very reliable measure of lower body power and has been one of the most frequently used tests for monitoring neuromuscular status in individual and team sports [43,44,45,46,47]. To further enhance the scope of assessment, we introduced the Countermovement Jump with Arms Thrust (CMJAT). Despite extensive research into the impacts of arm swing and countermovement on jump performance, the combined influence of these strategies on joint work and jump performance remains unexplored.

Regarding the placement of the sensor, the G sensor comes with a highly secure belt that is fastened with Velcro and buttons around the target area, this belt comes with a small pouch which is firmly attached to the target location of the sensor (based on the protocol selected); thus, it prevents the sensor from drifting during intensive tasks like jumping. Moreover, the software can detect if any sensor drifting occurred as the data collection automatically stops if the sensor is not placed in the exact location required by the selected protocol. A link to the website of the manufacturer has been provided to showcase the parts that come with the sensor (https://www.btsbioengineering.com/products/g-walk/, accessed on 12 August 2023). The protocols for walking and jumping are discussed in detail in the sections below. 

**A—Walking**

The sensor was placed on the L5-S1 vertebrae. The participant was then instructed to walk in a straight line using their natural speed to allow the execution of at least 5 complete gait cycles (>7 m) before making a change of direction. Before each change of direction, the participant was instructed to stop for a minimum of 1 s, turn around towards the new direction and take a pause of at least 1 s before starting to walk again.

The following parameters were selected from the measurements obtained through the walking protocol:

*1*— *Spatiotemporal Parameters-Global Analysis*

- Cadence (steps/min): number of steps taken by the participant in one minute;
- Speed (m/s): average walking speed;
- Stride length (m): average value of distances between each initial contact and the next one of the same sides;
- % Stride length (% height): Stride length normalized over the height of the subject.

*2*— *Stance Phase*

- This includes all the steps taken by the patient during the trial showcased by initial contact and toe off (thus showcasing the symmetry between right and left steps, and the symmetry between steps taken on each side, respectively.

*3*— *Gait Cycle*

**Figure 1:**
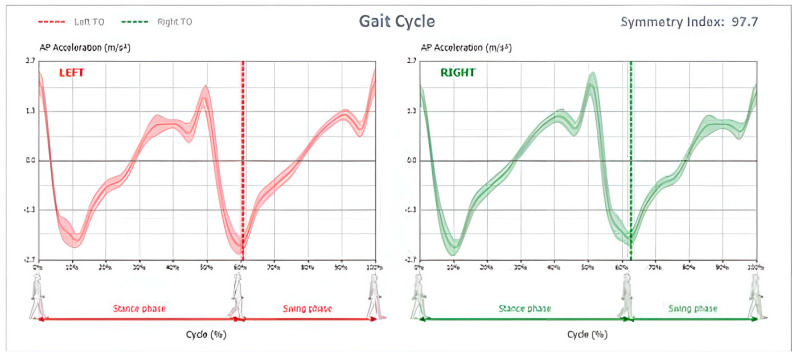
Symmetry Index shown to compare the curve of anteroposterior acceleration between left (red) and right (green) gait cycles. The light area around the line represents the normative ranges for this subject based on their demographic data.

- Different graphs representing left and right gait cycles starting with stance phase, shown via initial contact (0%), and the next initial contact on the same foot (100%), in addition to toe off (represented using a dotted line) to signal the start of the stride phase;
- Symmetry index, which is the percentage of symmetry between the curve of anterior/posterior acceleration during left and right gait cycles, the maximum value of 100 represents ideal symmetry throughout walking, this is displayed in Figure 1.

*4*— *Single Support Phases*

**Figure 2:**
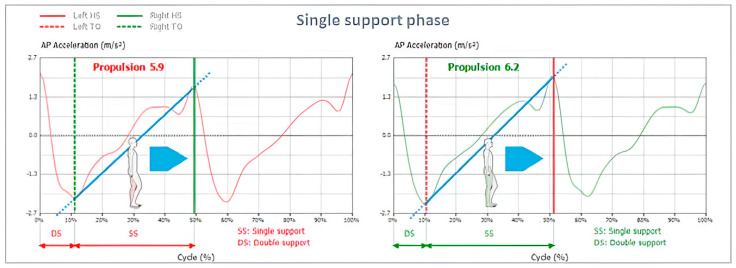
Propulsion during the single support phase is represented by the blue line. The slope indicates the symmetry between both sides where a more vertical line showcases better symmetry between left (red) and right (green) lines.

**Figure 3:**
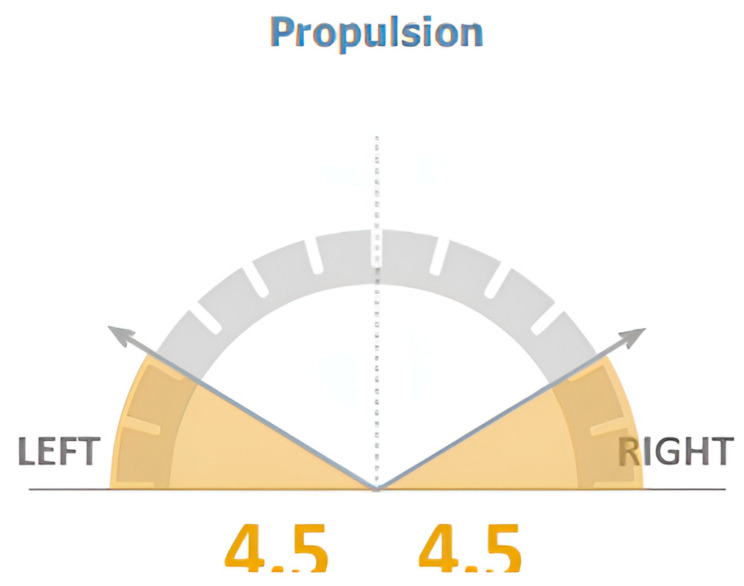
Propulsion Index calculated for left and right-side gait cycles.

- Propulsion (represented using a blue line), in addition to propulsion index (represented using the slope of the blue line) where more “vertical” lines indicate higher propulsion indices, and thus, better propulsion symmetry between both sides; this is shown in Figure 2 and Figure 3.

*5*— *Pelvic Angles*

**Figure 4:**
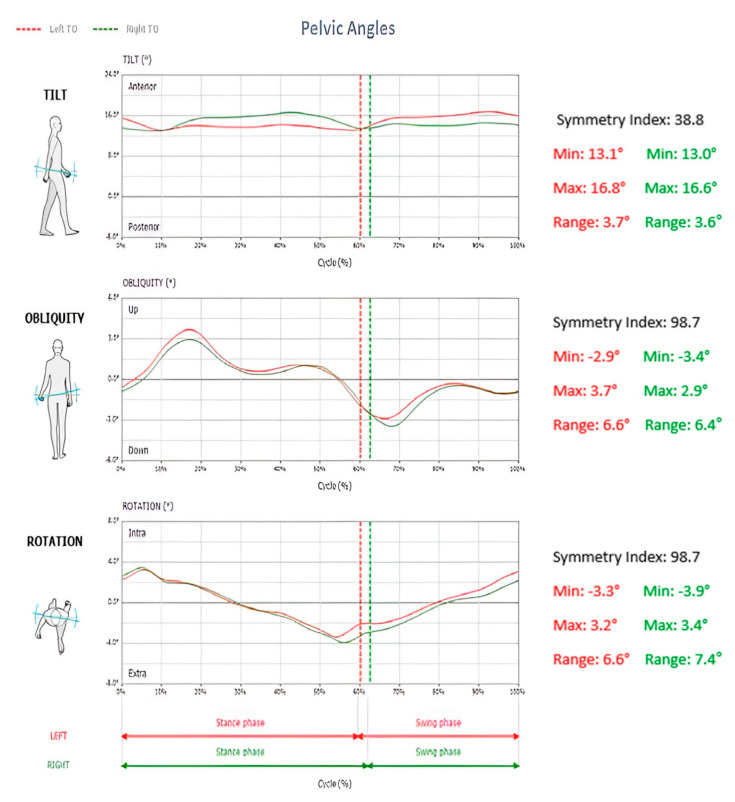
Pelvis Tilt, Obliquity, and Rotation measurements as shown in the generated report. The gray band indicates normative ranges for this subject based on their demographic data.

- Tilt: positive angular values indicate an anterior tilt of the pelvis, while negative angular values indicate a posterior tilt of the pelvis (sagittal plane movement);
- Obliquity: negative angular values indicate DOWN condition, while positive angular values indicate UP position for the considered side (frontal plane movement);
- Rotation: negative angular values indicate pelvis internally rotated, while positive angular values indicate a pelvis externally rotated (transverse plane movement);
- Relative symmetry index and minimum and maximum angles were shown for each of the 3 pelvic parameters mentioned above. All pelvic parameters are seen in Figure 4.

**B—Jumping**

All participants received a visual demonstration of the upcoming task, followed by a practice round to ensure correct performance prior to the actual collection of the data. The sensor was placed on L5-S1 vertebrae, and the upcoming jump was explained in detail to each participant prior to acquisition.

The jumping protocol covers 6 kinds of jumps. The following two were selected for this study:

*1*- *Countermovement Jump (CMJ)*

**Figure 5:**
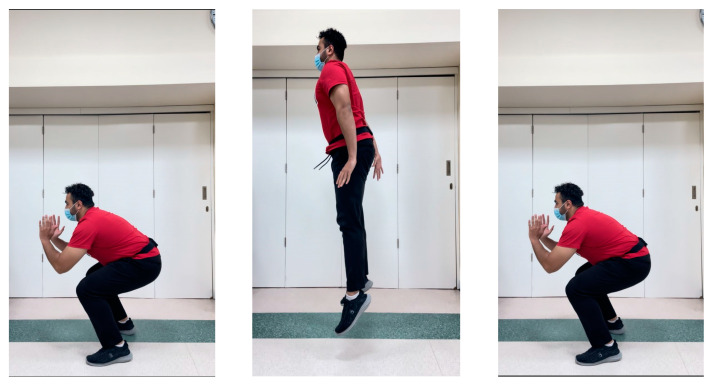
General sequence and belt location during the countermovement jump (CMJ). Note: herein, the volunteers hands were positioned out of the way as to not cover the belt for demonstration purposes only.

- The participant begins the test in upright position with their hands on the hips and their feet placed in line with the shoulders. They are then instructed to jump by performing a countermovement towards the downward direction and bending their knees by 90°. During the entire course of the test, the trunk should remain upright with hands near the hips (Figure 5).

*2*- *CMJ with Arms Thrust (CMJAT)*

**Figure 6:**
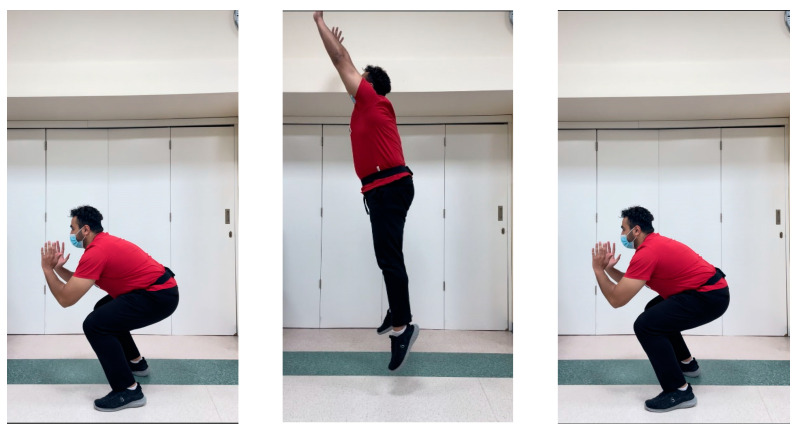
General sequence and belt location during the countermovement jump with arms thrust (CMJAT). Note: herein, the volunteers hands were positioned out of the way as to not cover the belt for demonstration purposes only.

- The participant begins the test in upright position with their hands on the sides and their feet placed in line with the shoulders. They are then instructed to jump by performing a countermovement towards the downward direction and bending their knees by 90°, with the help of using their arms as they extend them upwards. During the entire course of the test, the trunk should remain upright with arms and hands extending upwards in a thrust maneuver (Figure 6).

**Figure 7:**
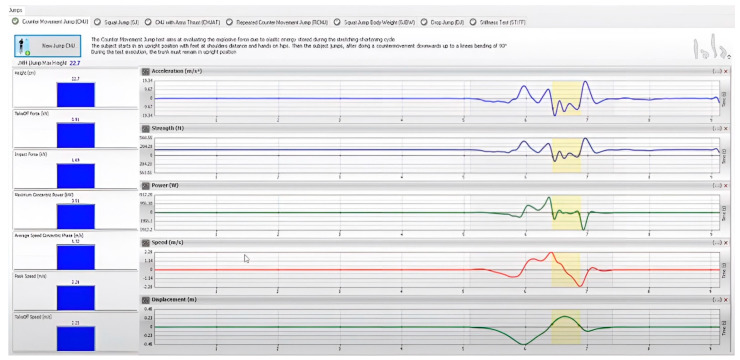
Jump parameters as shown in the G-Walk software.

The following information was computed for each jump and shown in Figure 7:

- Flight Height (cm);
- Take-Off Force (kN);
- Impact Force (kN);
- Take-Off Speed (m/s);
- Peak Speed (m/s);
- Average Speed Concentric Phase (m/s);
- Maximum Concentric Power (kW);
- Average Concentric Power (kW).

Each participant had 1 practice trial prior to data acquisition. Additionally, each trial was verbally explained and visually demonstrated by the data collector prior to data collection. Collection of all data occurred in one session with two-minute breaks given after each activity. Incorrect performance was pointed out and the trial was repeated to obtain accurate data based on the pre-set protocols.

### 2.2. Data Analysis

#### 2.2.1. Sample Size Determination

A Fisher Z transformation was used to estimate the sample size with the power level set at 0.80, the beta set at 0.20, and the alpha set at 0.05. The estimated sample size required for correlation was 74 participants; therefore, we enrolled a larger sample size of 100 to ensure external validity and to also strengthen the study.

#### 2.2.2. Statistical Analysis

Descriptive data are presented as the mean ± standard deviation. The Shapiro–Wilk test was used to determine the normality of the collected numerical variables. None of the numerical variables, however, satisfied the parametric assumptions; therefore, they are presented as the median and interquartile range (IQR). Spearman’s rank correlation coefficient was then performed, and the correlation coefficient (r) and *p*-values are reported to demonstrate the relationship between the head posture parameters and both the gait parameters and the jump parameters. The correlation coefficient ranges between −1 and 1, where a positive or negative sign indicates that the correlation between said variables have positive or negative relationships, respectively. The level of significance was set at 0.05. The interpretation table from Sugiyono et al. [48] was used as a reference to determine the strength of the correlations. The following key was used to interpret the correlations: where low correlations are described as “weak”, moderate is kept the same, and high/very high correlations are dubbed as “strong”.

## 3. Results

### 3.1. Participant Demographics and Characteristics

Participant characteristics are shown in Table 1. The Shapiro–Wilk test was used to test for the normality of the numerical variables reported; however, all the numerical variables did not follow the parametric assumptions, and thus were described using the median and interquartile range (Table 2, Table 3, Table 4 and Table 5).

### 3.2. Correlations between Variables

Several correlations were found between head posture parameters in terms of rotations and translations, displacements and gait parameters (Table 6). Firstly, moderate positive correlations were found between CVA and all gait parameters included with a *p* < 0.001 and r values that fall between 0.43 and 0.58 for cadence, speed, symmetry index, % left stride length (% height), % right stride length (% height), left propulsion index, right propulsion index, tilt symmetry index, tilt range left, tilt range right, obliquity symmetry index, obliquity range left, obliquity range right, rotation symmetry index, rotation range left, and rotation range right.

Moderate negative correlations were found between lateral translation of the head and speed (*r* = −0.46 *p* < 0.001), % left stride length (%height) (r = −0.42, *p* < 0.001), % right stride length (% height) (r = −0.51, *p* < 0.001) and tilt symmetry index (r = −0.51, *p* < 0.001). In addition, strong negative correlations were found between lateral translation of the head and cadence (r = −0.74, *p* < 0.001), symmetry index (r = −0.7, *p* < 0.001), and rotation symmetry index (r = −0.69, *p* < 0.001). The remaining variables showed weak negative correlations ranging between −0.06 and −0.36 (*p*-values ranging from 0.001 to 0.004) for left propulsion index, right propulsion index, tilt left range, tilt right range, obliquity symmetry index, obliquity right range, rotation left range, and rotation right range.

Various moderate negative correlations were found between AHT and cadence (r = −0.51, *p* < 0.001), speed (r = −0.44, *p* < 0.001), % left stride length (% height) (r = −0.48, *p* < 0.001), % right stride length (% height) (r = −0.46, *p* < 0.001), left propulsion index (r = −0.47, *p* < 0.001), tilt right range (r = −0.47, *p* < 0.001), rotation left range (r = −0.42, *p* < 0.001), and rotation right range (r = −0.41, *p* <0.001). In addition, strong negative correlations were found between AHT and tilt symmetry index (r = −0.62, *p* < 0.001), tilt left range (r = −0.81, *p* < 0.001), obliquity symmetry index (r = −0.75, *p* < 0.001), obliquity left range (r = −0.65, *p* < 0.001), and obliquity right range (r = −0.64, *p* < 0.001). Moreover, a weak negative correlation was found between AHT and right propulsion index (r = −0.39, *p* = 0.001) and rotation symmetry index (r = −0.27, *p* = 0.01).

Several moderate negative correlations were found between lateral angulation of the head and tilt symmetry index (r = −0.45, *p* < 0.001), obliquity symmetry index (r = −0.4, *p* < 0.001), and rotation symmetry index (r= −0.43, *p* < 0.001). In addition, weak negative correlations were found between lateral angulation and various other variables including cadence, speed, symmetry index, % left stride length (%height), % right stride length (%height), left propulsion index, right propulsion index, tilt left range, tilt right range, obliquity left range, obliquity right range, and rotation right range with r values ranging between −0.1 and −0.39 and *p*-values ranging from <0.001 to 0.011.

Multiple correlations were found between head posture parameters in terms of rotations and translations displacements and jump parameters (Table 7). Starting with CMJ, moderate positive correlations were found between CVA and flight height (r = 0.41, *p* < 0.001), average concentric speed phase (r = 0.41, *p* < 0.001), and average concentric power (r = 0.52, *p* < 0.001). In addition, multiple weak correlations were found between CVA and take-off force (r = 0.39, *p* < 0.001), take-off speed (r = 0.39, *p* < 0.001), peak speed (r = 0.38, *p* < 0.001), and maximum concentric power (r = 0.27, *p* = 0.03). As for lateral translation of the head, multiple negative correlations were found between it and flight height (r = −0.39, *p* < 0.001), take-off force (r = −0.4, *p* < 0.001), impact force (r = −0.48, *p* < 0.001), take-off speed (r = −0.43, *p* < 0.001), peak speed (r = −0.42, *p* < 0.001), average speed concentric phase (r = −0.42, *p* < 0.001), and average concentric power (r = −0.54, *p* < 0.001). Moreover, a weak negative correlation was found with maximum concentric power (r = −0.31, *p* < 0.003).

As for AHT coronal, moderate negative correlations were found with flight height (r = −0.44, *p* < 0.001), impact force (r = −0.55, *p* < 0.001), take-off speed (r = −0.49, *p* < 0.001), peak speed (r = −0.49, *p* < 0.001), average speed concentric phase (r = −0.57, *p* < 0.001), maximum concentric power (r = −0.41, *p* < 0.001) and average concentric power (r = −0.48, *p* < 0.001). In addition, a strong negative correlation was found with take-off force (r = −0.65, *p* < 0.001). As for lateral angulation, moderate negative correlations were found with peak speed, average speed concentric phase, and maximum concentric power (r = −0.4, *p* < 0.001) for all three variables. Moreover, weak negative correlations were found with the remaining variables with r values ranging from −0.37 to −0.39 (*p*-value of <0.001) (Table 7). 

As for CMJAT variables, weak positive correlations were found between CVA and impact force (r = 0.39, *p* < 0.001), average speed concentric phase (r = 0.39, *p* < 0.001), and maximum concentric power (r = 0.27, *p* < 0.001) (Table 8). On the other hand, moderate positive correlations were found with all the remaining variables with a *p*-value of < 0.001 and an r value ranging from 0.43 to 0.55. When it comes to lateral translation of the head (sagittal), weak negative correlations were found with flight height (r = −0.39, *p* < 0.001) and maximum concentric power (r= −0.31, *p* = 0.003). Moreover, moderate negative correlations were found with all the remaining CMJAT variables with a *p*-value of 0.001 and an r value ranging from −0.4 to −0.54. As for AHT, strong negative correlations were found with flight height (r = −0.7, *p* < 0.001) and take-off force (r = −0.65, *p* < 0.001). In addition, moderate negative correlations were found with all remaining CMJAT variables with a *p*-value of < 0.001 and an r value ranging from −0.48 to −0.6. Finally, for lateral angulation of the head, moderate negative correlations were found with impact force, take-off speed, and average speed concentric phase (r = 0.4, *p* < 0.001). In addition, weak negative correlations were found with the remaining CMJAT variables with a *p*-value of <0.001 and an r value ranging from −0.31 to −0.39 (Table 8).

## 4. Discussion

In this investigation, we examined the relationship between head postural displacements, specifically translations and rotations of the head, and gait and jump performance parameters. Our study’s primary hypothesis is supported by our findings in as much as we identified numerous statistically significant correlations between head posture displacements and many parameters related to gait and jump. Notably, we observed moderate to high correlations between CVA, lateral translation of the head, and AHT with all gait and jump parameters. Conversely, weak correlations were found between lateral angulation of the head and the gait and jump parameters, except for gait and pelvic symmetry index, where a moderate correlation was identified. These significant correlations emphasize the growing body of evidence demonstrating the significance of head posture related to functional performance measures.

It is noted that most studies assessing relationships between head posture and functional performance measures have rather exclusively assessed sagittal plane alignment only (e.g., AHT or the CVA) [9,12,13,14,49,50,51,52,53]. However, a unique contribution to the literature as determined in the present study is the statistically significant relationships between various gait and jump parameters and coronal head postures, including lateral head tilt and lateral head translation. It is also important to mention that these results are mainly limited to younger university students, who are known to have a forward head posture [51,52,53]. Importantly, however, our results indicate that coronal plane deviations of the head are likely also common and need to be evaluated.

### 4.1. Posture and Athletic Skills

We assessed two types of athletic skill activities: (1) jumping, which is correlated with athletic-based activities, and (2) gait, which is an integral part of daily ambulation. Regarding the jumping protocol utilized herein, the countermovement jump (CMJ) was selected because it is a simple, practical, valid, and very reliable measure of lower body power and has been one of the most used tests for monitoring neuromuscular status in individual, and team sports [43,44,45,46,47]. To further enhance the scope of the assessment, we introduced the countermovement jump with arms thrust (CMJAT). Despite extensive research into the impacts of arm swing and countermovement on jump performance, the combined influence of these strategies on joint work and jump performance remains largely unexplored. The simultaneous utilization of both arm swing and countermovement introduces the potential for additive, multiplicative, or even negative effects. As a result, a comprehensive understanding of the interaction between these two strategies, particularly concerning joint work, remains elusive. Given the close relationship between the upper limb and the cervical spine, we introduced the CMJAT alongside the CMJ.

The observed associations identified in our current investigation can be attributed to the significant impact of head posture on sensorimotor integration, which has been proven to influence the performance of specific tasks like walking and jumping [54]. Several studies have identified the adverse effects of head postural displacements on sensorimotor processing and integration [9,55,56,57]. Moustafa et al., for example, found college athletes with a forward head posture demonstrated both less efficient physical fitness performance as well as altered sensorimotor processing and integration as compared to athletes without a forward head position [58]. Thus, suboptimal performance of said tasks in individuals with abnormal head posture could be attributed to the fact that spinal dysfunction of any kind can negatively influence processing in the central nervous system because spinal dysfunction can lead to maladaptive central plastic changes, which in turn, likely results in abnormal responses subsequent to the altered input to the central nervous system [59,60,61,62,63]. This assumption has been supported by strong associations between parameters of sensorimotor integration and head posture.

Proper performance of a voluntary motor activity heavily relies on peripheral sensory input, wherein peripheral pathways transmit sensory data to the central motor cortex (M1). The posterior cingulate cortex and other regions of the parietal cortex, the supplementary motor area, the dorsal premotor cortex, the ventral premotor cortex, the basal ganglia, the cerebellum, the thalamus, the brainstem, and even the spinal cord itself are among the other regions where sensorimotor integration takes place, is influenced by it, and can ultimately change the motor output in M1. Therefore, abnormalities in normal afferent input processing can disrupt the processing of neural networks that are present in the cortical motor areas, thus leading to negatively impacted motor control [64,65,66,67]. This is supported by previous evidence which demonstrated that correcting the altered sagittal cervical spine aberrant alignment through structural rehabilitation (care specifically dedicated to improvement in alignment) led to more effective responses in several sensorimotor outcome metrics (balance, oculomotor control, head repositioning error) [68,69,70,71]. 

However, to the best of our knowledge, when it comes to jumping, no study has yet investigated the relationship between coronal plane alignment and jump parameters, which means there is a gap in the current evidence. Therefore, as performed in the current investigation, identifying the effects of head postural parameters in terms of rotation and translation measurements on biomechanical parameters related to various types of activities would seem essential to determine if the extent of said parameters affects different activity types differently, and to determine the degree of influence that head posture measurement differences had on a variety of athletic skills, including the jump variables measured herein.

### 4.2. Posture and Gait Asymmetries 

In the recent literature, adult spine deformity (ASD) categories have been investigated for their influence on and correlation with gait abnormalities; ASD categories include thoracic hyperkyphosis, anterior sagittal balance of C7-S1, decreased distal lumbar curve and pelvic retro-version, and coronal scoliosis > 20° [72,73,74,75,76]. The majority of investigations have identified that, in adults with ASD, the spine alignment profiles in the sagittal plane have clear and significant correlations with gait endurance, gait kinematics, and gait asymmetry [72,73,74,75,76]. When it comes to coronal plane alignment, only limited evidence explores the relationship between coronal ASD (scoliosis) and gait abnormalities, indicating that adverse alignments in the coronal plane also lead to alterations in performance during walking [72,73]. 

Similarly, previous investigations have identified that anatomical leg length inequality (ALLI) has a direct effect on gait alterations and adopted strategies of asymmetry in both the sagittal and coronal planes [77,78,79]. In a systematic review of the literature, Khamis and Carmeli identified that ALLI > 1 cm was significantly associated with altered gait and that the asymmetry increased with the magnitude of ALLI [77]. However, authors have demonstrated that mild ALLIs (ALLI = 5 mm–1 cm) also alter gait parameters [75]. Specific to the head and neck, in a recent systematic review, Lin and colleagues [12] found that the evidence to support a relationship between forward head posture and altered gait kinematics was lacking, with few previous investigations available, and these studies did not address non-sagittal cervical postures. Problematically in regard to coronal plane head rotations and translations, the current authors were unable to identify relevant studies that have specifically investigated altered postures of the head and their effect on gait kinematics. Thus, our investigation and its findings appear to be unique. 

### 4.3. Study Limitations

Several limitations can be proposed for this study, to be addressed in future works. The population of choice was a young, adult, overall healthy, and symptom-free population; thus, the results obtained from investigating a population with current symptoms are unknown. Second, future investigations should seek to identify if postural alterations of the thoracic cage and the pelvis have the potential to affect the variables presented herein to a greater, lesser, or equal extent to that identified herein. Thirdly, future investigations could conduct a more detailed subgrouping analysis of the data herein, such as by comparing gender groups based on head postural displacement characteristics. Fourth, due to the limited amount of previous investigations on the topic of jump/gait and cervical spine alignment [12], we chose a simpler design in our current project to see if specific 3D static posture displacements of the cervical spine have an effect on the biomechanics of gait and jump. Future investigations could assess dynamic characteristics of the cervical spine during the gait and jump performance in order to identify if detailed cervical spine kinematics are influenced by initial cervical static posture and/or if they have unique influences on the biomechanics of these dynamic tasks. Finally, since this is a cross-sectional investigation, it is unknown whether improvements, via interventional trials, in head posture variables are able to improve the gait and dynamic tasks reported herein.

## 5. Conclusions

We identified statistically significant relationships between various gait and jump functional performance measures and 3D head posture displacements. Our results indicate that studies focusing exclusively on the sagittal plane alignment are not sufficient to capture all possible correlations existing between 3D posture and performance measures; thus, it is recommended that 3D postural assessment approaches, which are now easily performed due to recent advancements in technology [35,36], are routinely used for studies evaluating relationships between static body posture and functional performance measures. Head postures as assessed as translations and rotations relative to neutral alignment, have important implications to performance parameters related to gait and jumping. Future studies are needed to further understand these relationships. 

## Figures and Tables

**Table 1:** Descriptive data for the demographic variables are presented. The values are presented as mean and standard deviation (SD) for age, weight, height, and BMI (body mass index) in addition to the distribution of the sample in terms of gender and ethnicity.

Variable	N = 100
Age (years)	21.10 ± 1.70
Weight (kg)	67.77 ± 17.28
Height (cm)	166.68 ± 8.72
BMI (kg/m^2^)	24.27 ± 5.33
Gender (%)	
Male	27
Female	73
Race/ethnicity (*n*)	
Arab–Non GCC	51
Arab–GCC	31
Non-Arab	18

**Table 2:** Descriptive statistics of posture parameters as the median and interquartile range (IQR).

Head Posture Parameters	N = 100 Median (IQR)
CVA (°)	51.60 (46.60, 55.30)
Lateral translation head (cm)	3.79 (2.68, 5.03)
AHT (cm)	0.69 (0.18, 1.21)
Lateral angulation head (°)	10.32 (6.67,14.3)

Note: CVA, craniovertebral angle; AHT, anterior head translation.

**Table 3:** Descriptive statistics of spatiotemporal parameters reported as the median and interquartile range (IQR).

Gait Spatiotemporal Parameters	N = 100 Median (IQR)
Cadence	109.70 (105.30, 116.30)
Speed (m/s)	1.15 (0.98, 1.24)
Symmetry index	92.20 (80.90, 95.60)
% Left stride length (% height)	76 (66.70, 83.20)
% Right stride length (% height)	75.40 (67.70, 82.60)
Left propulsion index	5.50 (4.20, 7.50)
Right propulsion index	4.80 (3.90, 7.60)
Tilt symmetry index	47.10 (24.30, 77.90)
Tilt left range	4.30 (3.30, 5.40)
Tilt right Range	8.40 (3.70, 13.20)
Obliquity symmetry index	95.80 (84.80, 98.10)
Obliquity left range	7.50 (5.70, 10.40)
Obliquity right range	80.00 (5.80, 10.50)
Rotation symmetry index	96.70 (85.70, 98.10)
Rotation left range	10.30 (6.90, 13.30)
Rotation right range	10.50 (6.90, 13.70)

**Table 4:** Descriptive statistics of countermovement jump (CMJ) parameters reported as the median and interquartile range (IQR).

CMJ Parameters	N = 100 Median (IQR)
CMJ–flight height (cm)	17.20 (14.20, 24.30)
CMJ–take–off force (kN)	1.11 (0.92, 1.40)
CMJ–impact force (kN)	1.35 (1.05, 1.69)
Take–off speed (m/s)	2.12 (1.85, 2.48)
CMJ–peak speed (m/s)	2.24 (1.99, 2.58)
CMJ–average speed concentric phase (m/s)	1.23 (1.08, 1.41)
CMJ–maximum concentric power (kW)	2.19 (1.67, 2.94)
CMJ–average concentric power (kW)	1.02 (0.85, 1.41)

**Table 5:** Descriptive statistics of countermovement jump with arms thrust (CMJAT) jump parameters reported as the median and interquartile range (IQR).

CMJAT Parameters	N = 100 Median (IQR)
CMJAT–flight height (cm)	19.60 (15.90, 27.70)
CMJAT–take–off force (kN)	1.15 (0.95, 1.55)
CMJAT–impact force (kN)	1.45 (1.11, 1.95)
CMJAT–take–off speed (m/s)	2.15 (1.94, 2.45)
CMJAT–peak speed (m/s)	2.27 (2.09, 2.53)
CMJAT–average speed concentric phase (m/s)	1.23 (1.12, 1.39)
CMJAT–maximum concentric power (kW)	2.11 (1.76, 3.34)
CMJAT–average concentric power (kW)	1.08 (0.89, 1.53)

**Table 6:** Spearman correlation coefficient (r) and *p*-values (*p*) between CVA, lateral translation head sagittal, AHT coronal, and lateral angulation head (first row), and each of the gait parameters (first column).

Gait Parameters	CVA		Lateral Translation Head		AHT		Lateral Angulation Head	
	r	*p*	r	*p*	r	*p*	r	*p*
Cadence	0.51 *	<0.001	−0.74 *	<0.001	−0.51 *	<0.001	−0.27 *	0.006
Speed (m/s)	0.52 *	<0.001	−0.46 *	<0.001	−0.44 *	<0.001	−0.37 *	<0.001
Symmetry index	0.54 *	<0.001	−0.70 *	<0.001	0.02	0.854	−0.31 *	0.003
%Left stride length (% height)	0.43 *	<0.001	−0.42 *	<0.001	−0.48 *	<0.001	−0.31 *	0.003
%Right stride length (% height)	0.51 *	<0.001	−0.51 *	<0.001	−0.46 *	<0.001	−0.32 *	0.01
Left propulsion index	0.54 *	<0.001	−0.36 *	0.001	−0.47 *	<0.001	−0.36 *	<0.001
Right propulsion index	0.55 *	<0.001	−0.29 *	0.015	−0.39 *	0.001	−0.39 *	<0.001
Tilt symmetry index	0.57 *	<0.001	−0.51 *	<0.001	−0.62 *	<0.001	−0.45 *	<0.001
Tilt left range	0.58 *	<0.001	−0.21 *	0.004	−0.81 *	<0.001	−0.37 *	<0.001
Tilt right range	0.54 *	<0.001	−0.23 *	0.004	−0.47 *	<0.001	−0.26 *	0.011
Obliquity symmetry index	0.56 *	<0.001	−0.06 *	<0.001	−0.75 *	<0.001	−0.40 *	<0.001
Obliquity Left Range	0.45 *	<0.001	−0.49 *	<0.001	−0.65 *	<0.001	−0.26 *	0.011
Obliquity right Range	0.54 *	<0.001	−0.25 *	0.005	−0.64 *	<0.001	−0.25 *	0.011
Rotation symmetry Index	0.52 *	<0.001	−0.69 *	<0.001	−0.27 *	0.010	−0.43 *	<0.001
Rotation left range	0.46 *	<0.001	−0.33 *	0.001	−0.42 *	<0.001	−0.10	0.3
Rotation right range	0.46 *	<0.001	−0.24 *	0.005	−0.41 *	<0.001	−0.26 *	0.011

* Indicates a statistically significant correlation (*p* < 0.05).

**Table 7:** Spearman correlation coefficient (r) and *p*-values (*p*) between CVA, lateral translation head, AHT, and lateral angulation head (first row), and each of the CMJ parameters (first column).

Physical Performance Skills	CVA		Lateral Translation Head		AHT		Lateral Angulation Head	
	r	*p*	r	*p*	r	*p*	r	*p*
CMJ–flight height (cm)	0.41 *	<0.001	−0.39 *	<0.001	−0.44 *	<0.001	−0.37 *	<0.001
CMJ–take-off force (kN)	0.39 *	<0.001	−0.40 *	<0.001	−0.65 *	<0.001	−0.38 *	<0.001
CMJ–impact force (kN)	0.24	0.052	−0.48 *	<0.001	−0.55 *	<0.001	−0.39 *	<0.001
Take-off speed (m/s)	0.39 *	<0.001	−0.43 *	<0.001	−0.49 *	<0.001	−0.38 *	<0.001
CMJ–peak speed (m/s)	0.38 *	<0.001	−0.42 *	<0.001	−0.49 *	<0.001	−0.40 *	<0.001
CMJ–average speed concentric phase (m/s)	0.41 *	<0.001	−0.42 *	<0.001	−0.57 *	<0.001	−0.40 *	<0.001
CMJ–maximum Concentric Power (kW)	0.27 *	0.03	−0.31 *	0.003	−0.41 *	<0.001	−0.40 *	<0.001
CMJ–average concentric power (kW)	0.52 *	<0.001	−0.54 *	<0.001	−0.48 *	<0.001	−0.39 *	<0.001

* Indicates a statistically significant correlation (*p* < 0.05).

**Table 8:** Spearman correlation coefficient (r) and *p*-values (*p*) between CVA, lateral translation head sagittal, AHT coronal, and lateral angulation head (first row), and each of the CMJAT parameters (first column).

CMJAT	CVA		Lateral Translation Head		AHT		Lateral Angulation Head	
	r	*p*	r	*p*	r	*p*	r	*p*
CMJAT–flight height (cm)	0.55 *	<0.001	−0.39 *	<0.001	−0.70 *	<0.001	−0.39 *	<0.001
CMJAT–take-off force (kN)	0.43 *	<0.001	−0.40 *	<0.001	−0.65 *	<0.001	−0.38 *	<0.001
CMJAT–impact force (kN)	0.39 *	<0.001	−0.48 *	<0.001	−0.55 *	<0.001	−0.40 *	<0.001
CMJAT–take-off Speed (m/s)	0.44 *	<0.001	−0.43 *	<0.001	−0.50 *	<0.001	−0.40 *	<0.001
CMJAT–peak speed (m/s)	0.48 *	<0.001	−0.42 *	<0.001	−0.55 *	<0.001	−0.39 *	<0.001
CMJAT–average Speed concentric phase (m/s)	0.39 *	<0.001	−0.42 *	<0.001	−0.60 *	<0.001	−0.40 *	<0.001
CMJAT–maximum concentric power (kW)	0.27 *	0.03	−0.31 *	0.003	−0.48 *	<0.001	−0.31 *	0.003
CMJAT–average concentric power (kW)	0.52 *	<0.001	−0.54 *	<0.001	−0.60 *	<0.001	−0.31 *	0.003

* Indicates a statistically significant correlation (*p* < 0.05).

## Data Availability

The datasets analyzed in the current study are available from the corresponding author upon reasonable request.
